# Electrochemical Properties of Rutile TiO_2_ Nanorod Array in Lithium Hydroxide Solution

**DOI:** 10.1186/s11671-016-1662-8

**Published:** 2016-10-06

**Authors:** Yan Yu, Dan Sun, Haibo Wang, Haiyan Wang

**Affiliations:** 1 Suzhou Polytechnic Institute of Agriculture, Suzhou, Jiangsu 215008 People’s Republic of China; 2 Institute of Chemical Power Sources & College of Physics, Optoelectronics and Energy, Soochow University, Suzhou, Jiangsu 215006 People’s Republic of China; 3 College of Chemistry and Chemical Engineering, Central South University, Changsha, 410083 People’s Republic of China

**Keywords:** Aqueous Li-ion batteries, Anode, Rutile TiO_2_, Nanorod array

## Abstract

In this paper, rutile TiO_2_ nanorod arrays are fabricated by a template-free method and proposed as a promising anode for aqueous Li-ion battery. The as-prepared TiO_2_ nanorod arrays exhibited reversible Li-ion insertion/extraction ability in aqueous LiOH electrolyte. Moreover, galvanostatic charge/discharge test results demonstrated that the reversible capacity of TiO_2_ nanorods could reach about 39.7 mC cm^−2^, and 93.8 % of initial capacity was maintained after 600 cycles at a current density of 1 mA cm^−2^ (=240 C rate), indicating excellent cycling stability and rate capability.

## Background

The continuously increasing demand for electronic devices, particularly the large-scale energy storage system (ESS) and electric vehicles (EVs), has forced researchers to investigate new classes of materials for replacement of the conventional Li-ion batteries (LIBs) and Ni-MH batteries [1]. In the mid of 1990s, a new type of Li-ion battery with aqueous electrolyte proposed by L. Wu et al. has intrigued many researchers due to its inherent safety, low-cost, and similar system as LIBs [2]. Compared with non-aqueous LIBs, the safety problem of aqueous Li-ion batteries (ALIBs) is fundamentally resolved, the ion conductivity of the electrolyte is enhanced by several magnitudes, and the rigorous assembly conditions are avoided, so the cost is greatly reduced [3, 4]. Unfortunately, ALIBs exhibited a poor cycling performance because of the complicated Li insertion/extraction process in aqueous solution, which would aggravate the dissolution of active materials and deterioration of crystal structure [3, 4]. Moreover, the decomposition of water also accelerates the fading of capacity and limits the working voltage of ALIBs.

Recently, the working voltage of ALIBs has been obviously improved using “water-in-salt” electrolyte, which attracts more and more researcher focusing on ALIBs [5, 6]. To obtain high-performance ALIB, advanced electrode materials are indispensable. Many strategies have been attempted to improve the electrochemical properties [3, 4], such as surface coating with conductive polymer or high-quality carbon, preparation of porous active materials [7], and fabrication of nanostructured active materials (e.g., nanoflake, nanotube, nanowire, and so on.) [8–14] Among these strategies, fabricating nanostructured electrodes demonstrate unique advantages over others [11, 12]. It is generally believed that nanostructured materials play an important role in electrochemical performance because of the high-specific surface area and short diffusion path which could enhance the electrochemical behavior of the applied battery [8, 14, 15].

In recent years, titanium dioxide (TiO_2_) has attracted considerable academic and practical interests [10, 16]. Its semi-conducting properties together with the excellent chemical stability make it an excellent candidate for solar cells, electrochromic devices, and rechargeable batteries [17–19]. In particular, nanostructured TiO_2_ for non-aqueous LIB anode has been extensively studied [20]. Recently, Manickam et al. proved that Li_x_TiO_2_ is electrochemically reversible in LiOH aqueous solution [21]. Reiman et al. demonstrated the reversibility of Li_*x*_TiO_2_ in aqueous solution with the aid of Dahn’s calculation of the HER potential vs. Li^+^/Li and fabricated TiO_2_ nano-film, which showed superior Li-ion storage properties [22]. Wu et al. prepared TiO_2_ nano-film via anodic electrodeposition method and obtained the diffusion coefficient through CVs method [14]. Note that the abovementioned TiO_2_ as anode for ALIBs are all concentrated on anatase phase. As we know, the phase plays an important role on the electrochemical properties [23]. Moreover, the one-dimensional nanosized materials (such as nanorod, nanotube, nanofiber, and so on [11, 12, 16, 24]) with faster kinetics and higher rate capability due to the large surface-to-volume ratio are attracting more and more attention [11].

In the present work, we first reported another phase of TiO_2_ (rutile phase) as anode for ALIBs. Rutile TiO_2_ nanorod array was deposited on fluorine-doped tin oxide (FTO) substrate via a template-free strategy, and the electrochemical performance of the as-synthesized TiO_2_ nanorod array in LiOH electrolyte was investigated in detail. It is interesting to note that the rutile TiO_2_ nanorod array exhibited excellent superior rate capability and excellent cycling stability, making it a promising anode candidate for ALIBs.

## Methods

In a typical synthesis, as a similar process described in the literature [16], 10 mL of toluene was mixed with 1 mL of 1 M titanium tetrachloride in toluene (97 % Aldrich) and 0.1 mL of tetrabutyl titanate in a Teflon-lined stainless steel autoclave (50 mL). The mixture was stirred at ambient conditions for 1 min followed by the addition of 1 mL of hydrochloric acid (37 wt %). After stirring for another 5 min, one piece of FTO glass substrate (3 cm × 1 cm) was placed at an angle against the wall of the Teflon liner. The FTO glass was initially cleaned by sonication in acetone, subsequently immersed in a 1 M NaOH solution for 10 min, and finally rinsed with ethanol and deionized water. Solvothermal synthesis was conducted at 180 °C for 2 h in an electric oven. After that, the autoclave was cooled to room temperature for approximately 70 min. Then the TiO_2_/FTO substrate was washed thoroughly with ethanol and allowed to dry under room temperature.

The crystal structure of the as-prepared film was examined by X-ray diffraction (XRD). The XRD patterns were recorded in a Bruker-AXS Micro-diffractometer (D8 ADVANCE) with Cu Kα radiation (*λ* = 1.5406 Å) from 10° to 70° at a scanning speed of 0.33° min^−1^. X-ray tube voltage and current were set at 40 kV and 40 mA, respectively. Morphological information was attained from scanning electron microscopy (SEM; HITACHI S-4800).

For electrochemical studies, electrical contact was made to some exposed FTO at the edge of the TiO_2_-coated slide, and the area of the TiO_2_ is 1 cm^2^. Conventional cyclic voltammetry (CV) and galvanostatic charge/discharge of the electrode were characterized by using a three-electrode cell in which an Hg/HgO and Pt sheet electrode were used as reference electrode and counter electrode, respectively. De-oxygenated 1 M aqueous LiOH solution was used as electrolyte. All electrochemical studies were conducted using CHI440a electrochemical station at room temperature.

## Results and Discussion

**Fig. 1:**
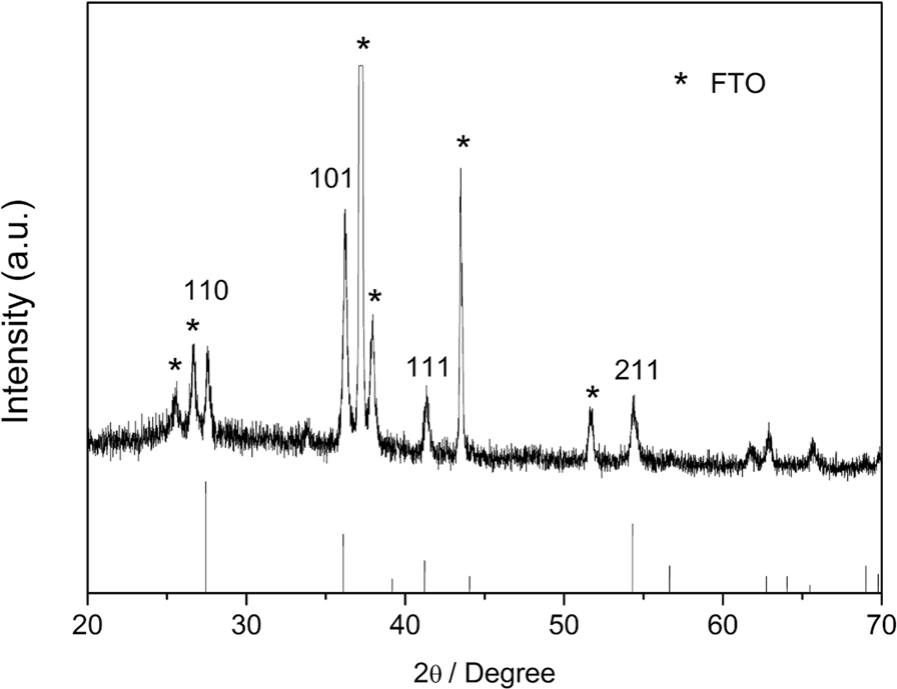
XRD patterns of rutile TiO_2_ nanorod array

**Fig. 2:**
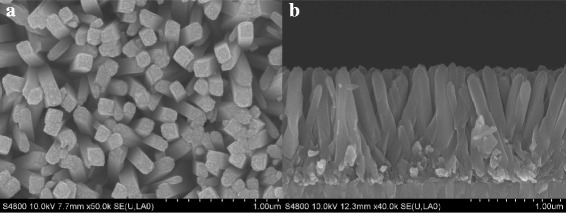
SEM images of rutile TiO_2_ nanorod array film grown on FTO substrate: **a** top-view SEM image, **b** cross-sectional SEM image

XRD pattern of the as-prepared sample is displayed in Fig. 1. As can be seen, the diffraction peaks, except for those with the asterisk which are due to the FTO substrate, could be well indexed to the tetragonal rutile TiO_2_ phase (JCPDS No. 21-1276) [16]. That is, rutile TiO_2_ could be deposited on the FTO substrate via a solvothermal process. The SEM images of top view and cross-sectional view of a typical TiO_2_ nanorod array sample are shown in Fig. 2. The images demonstrate that the surface of the FTO substrate is covered uniformly with aligned TiO_2_ nanorods. The top surface of the nanorods appears to be flat tetragonal crystallographic planes while the side surface is smooth. Nanorod arrays are nearly perpendicular to the FTO substrate. These results are consistent with the morphology of rutile nanorod arrays reported recently [16] and indicate that the TiO_2_ nanorod arrays have been successfully grown on FTO substrate. As determined from SEM images, the average diameter and length of nanorods are around 100 nm and 1.2 μm, respectively. It is well known that the application of 1D nanostructure in electrode design could provide high surface area and short ion path length, leading to higher capacities at high charge/discharge rates [11].

**Fig. 3:**
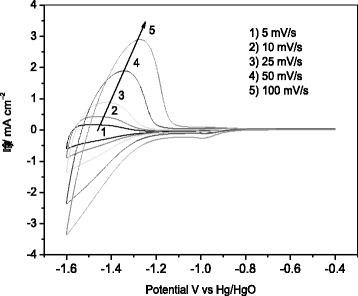
CV curves of TiO_2_ nanorod array between −0.40 and -1.60 V (vs. Hg/HgO) with different scanning rates in 1 M LiOH solution

CV curves of TiO_2_ nanorod array between −0.40 and −1.60 V (vs. Hg/HgO) with different scanning rates (5, 10, 25, 50, and 100 mV/s) in 1 M LiOH solution are shown in Fig. 3. Clearly, CV curves demonstrate a good Li-ions insertion/extraction reversibility of TiO_2_ nanorod array in LiOH solution. With the increase of scanning rate, the potential separation between the cathodic peak and anodic peak of TiO_2_ nanorod array become more and more larger, suggesting the increased polarization. The CV results at various scanning show the linear relationship (inset of Fig. 3) between the peak current and the square root of scan rate in anodic processes. The good linearity of anodic peaks reveals that the Li-ions insertion into the sample is a semi-diffusion controlled process, which can be treated as a quasi-reversible system with good reversibility [12, 14, 25–27].

**Fig. 4:**
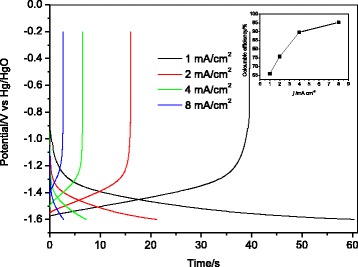
Galvanostatic charge/discharge curves of TiO_2_ nanorod array between −0.40 and −1.60 V (vs. Hg/HgO) at different current densities in 1 M LiOH solution (*inset* is the coulombic efficiency)

Galvanostatic charge/discharge curves of TiO_2_ nanorod array at different current densities in LiOH solution are displayed in Fig. 4. A charge/discharge plateau around −1.4 V vs. Hg/HgO is observed. Table 1 summarizes the specific capacity of TiO_2_ nanorod array at different current densities. As seen, the specific capacity gradually decreases with the increasing of charge/discharge current density. However, the coulombic efficiency shows a reverse change. As we know, the decomposition of H_2_O during the charge/discharge capacity is the main reason for the low coulombic efficiency of ALIB [3, 7]. Although the Li insertion potential of TiO_2_ is relatively low, it is possible to achieve the Li-ion insertion/extraction of TiO_2_ before the hydrogen evolution reaction (HER) by tuning the electrolyte systems and the current collectors. In this work, we used the LiOH solution, rather than neutral Li_2_SO_4_ or LiNO_3_ as electrolyte, in which the HER would be well suppressed. In addition, increasing the current density could further reduce the decomposition of water. At a low current density, the HER is serious, which results in a low coulombic efficiency (65.8 % for 1 mA cm^−2^). On increasing the current density, the polarization of HER increases and reaction time shortens, resulting in a much higher coulombic efficiency (95.2 % for 8 mA cm^−2^).

**Table 1:** Specific capacity of TiO_2_ nanorod array tested in Fig. 3

Current density (mA cm^−2^)	Charge capacity (mC cm^−2^)	Discharge capacity (mC cm^−2^)
1	60.3	39.7
2	42.4	32.1
4	29.1	26.1
8	23.1	22.0

Charge/discharge between −0.2 and −1.60 V vs. Hg/HgO in 1 M LiOH solution at 25 °C

**Fig. 5:**
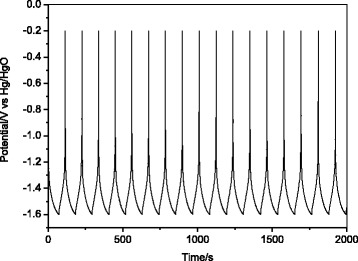
Galvanostatic charge/discharge curves of TiO_2_ nanorod array between −0.20 and −1.60 V (vs. Hg/HgO) at 1 mA cm^-2^ in 1 M LiOH aqueous solution

A constant current charge/discharge measurement of TiO_2_ nanorod array at 1 mA cm^-2^ was performed and some of charge/discharge curves are shown in Fig. 5. This result is significant when we realize that it is for a macroscopically planar electrode surface, so that much larger current densities can be expected for electrodes in which the same film thickness is distributed over a microscopically roughened current collector as in modern battery electrodes. Accordingly, since the actual TiO_2_ film thickness here was only 1.2 μm, we can predict that an external current density of nearly 1 A cm^-2^ should be possible for a typical battery electrode thickness of 120 μm.

**Fig. 6:**
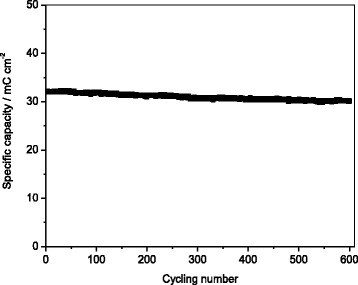
Cycling performance of TiO_2_ nanorod array at a current density of 1 mA cm^-2^ between −0.2 and −1.6 V (vs. Hg/HgO) in 1 M LiOH solution

Figure 6 displays the capacity vs. cycle number data for the TiO_2_ nanorod array electrode. These data were obtained from galvanostatic charge/discharge measurement over the potential range of −0.2~−1.6 V at a current density of 1 mA cm^-2^. The initial discharge capacity obtained over this potential window was 39.5 mC cm^−2^. After 600 cycles, the discharge capacity only decreased by 7 % of the original value, indicating the superior cycling stability of TiO_2_ nanorod array in LiOH solution. This represents another unique advantage of the nanostructured electrode.

Generally, low utilization of rutile TiO_2_ nanorod was demonstrated in aqueous solution in comparison with that observed in organic electrolyte due to the narrow electrochemical stability window of aqueous solution. Anyway, this research indicates that Li ions are partially electro-reversible in rutile TiO_2_, and the unique nanostructure facilitates the electrochemical behavior significantly.

## Conclusions

Rutile TiO_2_ nanorod array growing on FTO substrate was obtained successfully. CV results showed that Li-ion intercalation into/extraction from rutile TiO_2_ electrode can be occurred in LiOH solution. The TiO_2_ nanorod array demonstrated a good rate capability up to 39.7 mC cm^−2^ at 1 mA cm^−2^, much higher than that in organic electrolyte. Moreover, excellent cycling stability with only 7 % capacity loss after 600 cycles was observed for TiO_2_ nanorod array electrode. This work implies that nanostructured TiO_2_ array could be used as a promising anode for advanced aqueous Li-ion battery.

## Abbreviations

ALIBs: Aqueous Li-ion batteries
ESS: Energy storage system
EVs: Electric vehicles
LIBs: Li-ion batteries
TiO_2_: Titanium dioxide
